# Automated synthesis of [^18^F]DCFPyL via direct radiofluorination and validation in preclinical prostate cancer models

**DOI:** 10.1186/s13550-016-0195-6

**Published:** 2016-05-04

**Authors:** Vincent Bouvet, Melinda Wuest, Hans-Soenke Jans, Nancy Janzen, Afaf R. Genady, John F. Valliant, Francois Benard, Frank Wuest

**Affiliations:** Department of Oncology, University of Alberta, 11560 University Avenue, Edmonton, AB T6G 1Z2 Canada; Department of Chemistry and Chemical Biology, McMaster University, Hamilton, Canada; Department of Radiology, University of British Columbia, Vancouver, Canada

**Keywords:** ^18^FDCFPyL, Positron emission tomography (PET), Automated radiosynthesis, Prostate-specific membrane antigen (PSMA), Prostate cancer

## Abstract

**Background:**

Prostate-specific membrane antigen (PSMA) is frequently overexpressed and upregulated in prostate cancer. To date, various ^18^F- and ^68^Ga-labeled urea-based radiotracers for PET imaging of PSMA have been developed and entered clinical trials. Here, we describe an automated synthesis of [^18^F]DCFPyL via direct radiofluorination and validation in preclinical models of prostate cancer.

**Methods:**

[^18^F]DCFPyL was synthesized via direct nucleophilic heteroaromatic substitution reaction in a single reactor TRACERlab FX_FN_ automated synthesis unit. Radiopharmacological evaluation of [^18^F]DCFPyL involved internalization experiments, dynamic PET imaging in LNCaP (PSMA+) and PC3 (PSMA−) tumor-bearing BALB/c nude mice, biodistribution studies, and metabolic profiling. In addition, reversible two-tissue compartmental model analysis was used to quantify pharmacokinetics of [^18^F]DCFPyL in LNCaP and PC3 tumor models.

**Results:**

Automated radiosynthesis afforded radiotracer [^18^F]DCFPyL in decay-corrected radiochemical yields of 23 ± 5 % (*n* = 10) within 55 min, including HPLC purification. Dynamic PET analysis revealed rapid and high uptake of radioactivity (SUV_5min_ 0.95) in LNCaP tumors which increased over time (SUV_60min_ 1.1). Radioactivity uptake in LNCaP tumors was blocked in the presence of nonradioactive DCFPyL (SUV_60min_ 0.22). The muscle as reference tissue showed rapid and continuous clearance over time (SUV_60min_ 0.06). Fast blood clearance of radioactivity resulted in tumor-blood ratios of 1.0 after 10 min and 8.3 after 60 min. PC3 tumors also showed continuous clearance of radioactivity over time (SUV_60min_ 0.11). Kinetic analysis of PET data revealed the two-tissue compartmental model as best fit with *K*_1_ = 0.12, *k*_2_ = 0.18, *k*_3_ = 0.08, and *k*_4_ = 0.004 min^−1^, confirming molecular trapping of [^18^F]DCFPyL in PSMA+ LNCaP cells.

**Conclusions:**

[^18^F]DCFPyL can be prepared for clinical applications simply and in good radiochemical yields via a direct radiofluorination synthesis route in a single reactor automated synthesis unit. Radiopharmacological evaluation of [^18^F]DCFPyL confirmed high PSMA-mediated tumor uptake combined with superior clearance parameters. Compartmental model analysis points to a two-step molecular trapping mechanism based on PSMA binding and subsequent internalization leading to retention of radioactivity in PSMA+ LNCaP tumors.

**Electronic supplementary material:**

The online version of this article (doi:10.1186/s13550-016-0195-6) contains supplementary material, which is available to authorized users.

## Background

Prostate cancer is among the most common malignancy in men in Western countries and accounts for the fifth leading cause of cancer-related death in men [1]. This situation clearly underscores prostate cancer as a highly frequent but still unmet medical challenge which continues to be the target of a significant proportion of current clinical research, including to a large extent the development of novel radiotracers for positron emission tomography (PET) imaging of prostate cancer [2, 3]. PET imaging of prostate cancer was successfully demonstrated with various ^11^C- and ^18^F-labeled choline analogs, as well as ^11^C-acetate for imaging cell membrane and fatty acid metabolism in prostate cancer [4, 5]. PET imaging of prostate cancer with [^18^F]FDG, the most widely used radiopharmaceutical for cancer imaging, gave mixed results. [^18^F]FDG-PET is not useful in the detection of primary organ-confined prostate cancer and local recurrences after radical prostatectomy, as well as in differentiating between post-operative scar and local recurrence. However, [^18^F]FDG uptake in prostate cancer was reported to correlate with prostate-specific antigen (PSA) level as a biomarker to measure tumor aggressiveness [6]. [^18^F]FDG-PET was also used to monitor therapeutic response of patients with androgen-independent disease [7, 8].

The aforementioned limitations of [^18^F]FDG-PET in connection with the importance of prostate cancer as a significant unmet clinical need drive current developments towards targeted molecular imaging of prostate cancer. One prominent class of agents that exemplifies the outcome of this effort includes radiotracers targeting the trans-membrane protein, prostate-specific membrane antigen (PSMA) [9]. PSMA is a highly promising biomarker for targeted prostate cancer imaging due to its elevated expression and up-regulation in poorly differentiated, metastatic, and androgen-independent carcinomas.

PSMA is a particularly important molecular target in prostate cancer patients with negative bone scans who are at high risk for metastatic disease. Moreover, PSMA is an ideal target for developing small-molecule radiopharmaceuticals which typically show fast blood clearance and low background activity [9]. Over the past decade, several urea-based and phosphoramidate peptidomimetic inhibitors of PSMA have been developed and labeled with positron emitters such as ^11^C, ^18^F, ^68^Ga, ^64^Cu, ^124^I, and ^86^Y [9]. Among these small-molecule PSMA-imaging agents, especially ^68^Ga-labeled compounds have been introduced into clinical applications for PSMA imaging in prostate cancer patients [10, 11]. Although ^68^Ga-labeled small-molecule PSMA inhibitors exhibit favorable clinical imaging properties, improved lesion detection could be further enhanced using ^18^F due to its lower positron energy and longer half-life, or improved retention due to the chemical nature of existing agents. This was recently demonstrated by the first comparative clinical study of ^18^F-labeled PSMA inhibitor [^18^F]DCFPyL with [^68^Ga]Ga-PSMA-HBED-CC [12].

Another challenge associated with ^68^Ga-labeled radiopharmaceuticals for clinical imaging is the less than optimal availability of ^68^Ga generators, especially with respect to their regulatory status in North America. Given the small quantity of ^68^Ga eluted from ^68^Ga generators (typically up to 1850 MBq), producing more than 1–3 doses per production batch is also challenging, which increases production costs.

However, despite the inherent advantages of ^18^F like the favorable physical half-life of 109.8 min and the convenient availability as cyclotron-produced [^18^F]fluoride at high specific activity, the production of ^18^F-labeled small-molecule PSMA inhibitors is complicated and often low yielding [13–20].

**Fig. 1:**
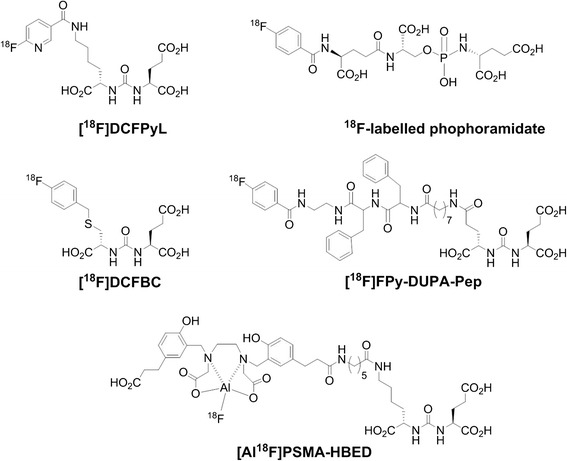
Structure of ^18^F-labeled small-molecule PSMA inhibitors

In all cases, radiosyntheses were accomplished via challenging prosthetic group-based multi-step synthesis routes or aluminum-[^18^F]fluoride acceptor chemistry. Prosthetic group-based radiosyntheses included preparation of 2,3,5,6-tetrafluorophenyl-6-[^18^F]fluoronicotinate ([^18^F]FPy-TFP), 4-[^18^F]fluorobenzyl bromide [^18^F]FBB) and *N*-succinimidyl-4-[^18^F]fluorobenzoate ([^18^F]SFB) as ^18^F-containing building blocks followed by bioconjugation chemistry through *N*-acylation and *S*-alkylation reactions, respectively (Fig. 1).

First clinical results demonstrated that [^18^F]DCFPyL is a promising radiotracer for PET imaging of prostate cancer and clear cell renal cell carcinoma. The promising clinical results stimulated efforts for the development of automated radiosyntheses based on more desirable direct radiofluorination reactions to simplify preparation and therefore to improve availability of [^18^F]DCFPyL as a radiopharmaceutical for PET imaging of PSMA.

Here, we describe a novel automated radiosynthesis of [^18^F]DCFPyL via a direct radiofluorination procedure in an automated synthesis unit (ASU) suitable for use under good manufacturing practice (GMP) guidelines. We also present preclinical data on the radiopharmacological profile of [^18^F]DCFPyL in PSMA+ LNCaP and PSMA− PC3 tumor xenografts which further confirmed the quality of the resulting radiotracer for targeted molecular imaging of PSMA in prostate cancer.

## Methods

All chemicals, reagents, and solvents for the synthesis and analysis were analytical grade. The amino acid precursors H-Glu(O^*t*^Bu)-O^*t*^Bu.HCl and H-Lys(Z)-O^*t*^Bu.HCl were purchased from EMD Millipore. The Bu_4_N^+^-HCO_3_^−^ solution and Chromafix (30-PS-HCO3, Macherey-Nagel) cartridges were purchased from ABX. All other chemicals and solvents were purchased from Sigma-Aldrich. All solvents were dried and/or distilled prior to utilization. ^1^H-NMR and ^13^C-NMR spectra were recorded on an Agilent/Varian Inova two-channel 400-MHz spectrometer, an Agilent/Varian Inova four-channel 500-MHz spectrometer, and an Agilent/Varian VNMRS three-channel 600-MHz spectrometer. Chemical shifts are given in parts per million (ppm) referenced to internal standards (s = singlet, bs = broad singlet, d = doublet, dd = doublet of doublet, ddd = doublet of doublet of doublet, t = triplet, M = multiplet, m = massif). Mass spectra were recorded using a Micromass ZABSpec Hybrid Sector-TOF by positive mode electrospray ionization. High resolution mass spectra (HRMS) were carried out on an Agilent Technologies 6220 oaTOF. Crude reaction mixtures were analyzed by TLC and HPLC. Thin-layer chromatography (TLC) was monitored using HF254 silica gel. HPLC analyses were performed on a semi-preparative Luna C18 column (100 Å, 10 μm, 250 × 10 mm) or Jupiter C12 (100 Å, 10 μm, 250 × 10 mm).

Both columns were connected to their corresponding guard columns (Phenomenex Nucleosil LUNA (II) RP C18 pre-column (5 μm, 50 × 10 mm) and Jupiter C12 pre-column (5 μm, 50 × 10 mm)). UV detection was performed at 210 and 254 nm. Radioactivity detection was achieved using a well-scintillation NaI (Tl) detector.

[^18^F]Fluoride was produced by the ^18^O(p,n)^18^F nuclear reaction through proton irradiation of enriched (98 %) ^18^O water (3.0 mL, ROTEM, Germany) using a TR19/9 cyclotron (Advanced Cyclotron Systems, Inc., Richmond, BC, Canada).

### Chemical synthesis

#### (*S*)-Di-*tert*.-butyl-(((2,5-dioxopyrrolidin-1-yl)oxy)carbonyl)-l-glutamate 2

One hundred fifty milligrams (0.507 μmol) of Glu(O^*t*^Bu)-O^*t*^Bu·HCl **1** was dissolved in 2 mL of freshly distilled CH_3_CN. Seventy microliters of Et_3_N (0.507 μmol) and 142 mg (0.554 μmol) of *N*,*N*′-disuccinimidyl carbonate was added to the solution. The reaction was stirred at 25 °C for 12 h and concentrated under reduced pressure. After re-solubilization in 5 mL of EtOAc and successive washing with 10 mL of 10 % citric acid and 10 mL of brine, the organic layer was dried over Na_2_SO_4_ and concentrated under reduced pressure to afford 197 mg of a pale yellow powder. This unpurified powder contained 90 % of desired compound **2** (yield 78 %). TLC: (EtOAc/hexane, 3/1): *R*_*f*_ = 0.7. ^1^H-NMR (400 MHz, CDCl_3_) δ: 1.46 (s, 9H), 1.50 (s, 9H), 1.94–2.14 (m, 1H), 2.10–2.21 (m, 1H), 2.24–2.44 (m, 2H), 2.83 (s, 4H), 4.23 (dt, 1H, J = 5.1 Hz, J = 7.7 Hz), 6.19 (d, 1H, J = 7.7 Hz). ^13^C-NMR (100.5 MHz, CDCl_3_) δ: 25.47, 27.59, 27.97, 28.00, 31.15, 54.78, 80.95, 83.09, 151.06, 169.60, 169.76, 171.95.

#### (*S*)-2-[3-((*S*)-1-*tert*.-Butylcarboxylate-(5-benzyloxycarbonylpentyl))ureido]-di-*tert*.-butyl pentanedioate 4

Compounds **4** and **5** and DCFPyL were previously synthesized [15, 16]. Herein, we describe an improved methodology. The yellow powder containing 90 % of compound **2** (197 mg, 90 % purity) was dissolved without further purification in 3 mL of CH_2_Cl_2_ containing 100 μL of Et_3_N and 187 mg (501 μmol) of μs-benzyloxycarbonyl-l-lysine *tert*.-butyl ester hydrochloride (H-Lys(Z)-O^*t*^Bu.HCl) **3**. The reaction mixture was stirred for 12 h at 25 °C, and progress of the reaction was monitored by TLC (CH_2_Cl_2_/MeOH 95/5). Upon completion, the reaction mixture was concentrated under reduced pressure and the residue was purified by column chromatography (CH_2_Cl_2_/MeOH 95/5) to afford 252 mg (92 %) of desired compound **4** as a clear gel. TLC: (CH_2_Cl_2_/MeOH 95/5): *R*_*f*_ = 0.2. ^1^H-NMR (600 MHz, CDCl_3_) δ: 1.16–1.24 (m, 1H), 1.24–1.30 (m, 1H), 1.35 (s, 9H), 1.37 (s, 9H), 1.38 (s, 9H), 1.39–1.47 (m, 2H), 1.48–1.56 (m, 1H), 1.61–1.69 (m, 1H), 1.69–1.77 (m, 1H), 1.93–2.01 (m, 1H), 2.13–2.25 (m, 2H), 3.02–3.15 (m, 2H), 4.22–4.28 (m, 1H), 4.28–4.33 (m, 1H), 4.99 (d, 1H, J = 12.5 Hz), 5.05 (d, 1H, J = 12.5 Hz), 5.37 (d, 1H, J = 8 Hz), 5.44 (s, 1H), 5.45 (d, 1H, J = 8.1 Hz), 7.20–7.24 (m, 1H), 7.24–7.30 (m, 4H). ^13^C-NMR (150.9 MHz, CDCl_3_) δ: 21.36, 26.98, 27.00, 27.05, 27.32, 28.33, 30.56, 31.61, 39.69, 51.84, 52.25, 65.46, 79.44, 80.58, 81.14, 126.93, 127.03, 127.43, 135.76, 155.66, 156.10, 171.33, 171.57, 171.89. *m/z* (ESI) C_32_H_51_N_3_O_9_ ([M + H^+^]) calcd. 622.4, found 622.4.

#### (*S*)-2-[3-((*S*)-5-Amino-1-*tert*.-butoxycarbonylpentyl)ureido]pentanedioic acid di-*tert*.-butyl ester 5 [15, 16]

A solution of compound **4** (220 mg, 0.354 μmol) in 3 mL of MeOH was stirred with 10 mg of Pd/C under H_2_ atmosphere for 12 h. The reaction was filtered through celite and concentration under reduced pressure afforded 164 mg (95 %) of compound **5** as a clear gel. ^1^H-NMR (600 MHz, CD_3_OD) δ: 1.45–1.47 (m,1H), 1.47 (s, 9H), 1.47–1.49 (m, 1H) 1.49 (s, 9H) 1.50 (s, 9H), 1.57–1.70 (m, 3H), 1.78–1.86 (m, 2H), 2.04–2.10 (m, 1H), 2.28–2.40 (m, 2H), 2.79–2.88 (m, 2H), 4.18 (dd, 1H, J = 5.1 Hz, J = 9.0. Hz), 4.22 (dd, 1H, J = 5.0 Hz, J = 8.9 Hz). *m/z* (ESI) C_24_H_45_N_7_O_7_ ([M + H^+^]) calcd. 488.3, found 488.3.

#### (*S*)-2-[3-((*S*)-1-Carboxy-5-[3-(6-fluoropyridine)carbonyl)amino)pentyl)ureido]-pentanedioic acid (DCFPyL) [15]

In a flame-dried flask, HBTU (156 mg, 410 μmol), DIPEA (72 μL, 410 μmol), and 6-fluoronicotinic acid **6** (58 mg, 410 μmol) were added to a solution of 100 mg (0.205 μmol) of compound **5** in 4 mL of distilled CH_2_Cl_2_. The reaction was stirred at 25 °C for 3 h under nitrogen, and progress of the reaction was monitored by TLC (hexane/ethyl acetate 1/1 *v*/*v*). Upon completion, the reaction mixture was concentrated under reduced pressure and the residue was purified by column chromatography (CH_2_Cl_2_/MeOH 97/3) to afford 97 mg (78 %) of *tert*.-butyl ester intermediate as a white powder. TLC: (EtOAc/hexane 1/1): *R*_*f*_ = *0.45*. ^1^H-NMR (600 MHz, CDCl_3_) δ: 1.39 (s, 9H), 1.41–1.46 (m, 19H), 1.51–1.63 (m, 2H), 1.64–1.71 (m, 1H), 1.73–1.80 (m, 1H), 1.80–1.86 (m, 1H), 1.98–2.08 (m, 1H), 2.25–2.39 (m, 2H), 3.33–3.42 (m, 1H), 3.50–3.59 (m, 1H), 3.69–3.77 (m, 1H), 4.45–4.24 (m, 2H), 5.59 (t, 1H, J = 9.5 Hz), 5.88 (t, 1H, J = 9.8 Hz), 7.00 (d, 1H, J = 8.1Hz), 7.81–7.90 (m, 1H), 8.41 ( t, 1H, J = 7.9 Hz), 8.81 (s, 1H). ^13^C-NMR (150.9 MHz, CDCl_3_) δ: 23.36, 27.81, 27.88, 27.95, 28.04, 28.66, 31.51, 32.43, 39.93, 53.12, 53.69, 80.72, 81.64, 82.54, 109.09 (d, J = 37.6 Hz), 128.56 (d, J = 4.3 Hz) 140.96 (d, J = 8.7 Hz), 147.95 (d, J = 13.1 Hz), 157.60, 164.86 164.87 (d, J = 244 Hz), 172.16, 172.31, 173.49. *m/z* (ESI) C_30_H_47_FN_4_O_8_ ([M + H^+^]) calcd. 611.3, found 611.3.

*Tert.*-butyl ester intermediate was treated with 6 mL of CH_2_Cl_2_/TFA (1/1 *v*/*v*) for 8 h at 25 °C, concentrated under reduced pressure, and purified by HPLC. HPLC purification was performed on a semi-preparative Jupiter C12 column (100 Å, 10 μm, 250 × 10 mm). The eluting solvent started with a 10/90 CH_3_CN/(water 0.5 % TFA) gradient for 5 min at a flow rate of 2 mL min^−1^ followed by a gradient from 10/90 to 70/30, *v*/*v*, for 25 min. Compound DCFPyL eluted at 14.5 min, and after solvent removal, 54 mg (67 %) of DCFPyL was isolated as a white powder. ^1^H-NMR (400 MHz, D_2_O) δ: 1.42–1.56 (m, 2H), 1.60–1.73 (m, 2H), 1.73–1.83 (m, 1H), 1.85–1.95 (m, 1H), 1.95–2.05 (m, 1H), 2.13–2.24 (dt, 1H, J_d_ = 20.6 Hz, J_t_ = 7.5 Hz), 2.51 (t, 2H, J = 7.2 Hz), 3.43 (t, 2H, 6.5 Hz), 4.25 (ddd, 2H, J = 9.4 Hz, J = 5.1 Hz, J = 9.2 Hz), 7.23 (d, 1H, J = 8.2 Hz), 8.31 (dt, 1H J_t_ = 8.2 Hz, J_d_ = 2.2 Hz), 8.57 (d, 1H, J = 2.2 Hz).^13^C-NMR δ (125.7 MHz, D_2_O): 23.26, 27.11, 28.56, 31.00, 31.55, 40.68, 53.51, 54.07, 109.05 (d, J = 37.8 Hz), 128.47 (d, J = 4.4 Hz) 140.87 (d, J = 8.1 Hz), 147.98 (d, J = 12.9 Hz), 160.26, 164.62 (d, J = 240.1 Hz), 168.16, 177.12, 178.05, 178.19. *m/z* (HRMS) C_18_H_22_FN_4_O_8_ ([M − H^+^]) calcd. 441.1427, found 441.1430.

#### 6-Trimethylammonium-nicotinic acid 2,3,5,6-tetrafluorophenyl ester triflate salt 8 (adapted from reference [21])

One gram (6.34 mmol) of 6-chloronicotinic acid 7; 1.1 g (6.5 mmol) of 2,3,5,6 tetrafluorophenol; and 1.31 g (6.34 mmol) of *N*,*N*′-dicyclohexylcarbodiimide (DCC) were stirred in dioxane (40 mL) for 5 h at 25 °C. Progress of the reaction was monitored by TLC (EtOAc/hexane 4/1). Upon completion, the reaction mixture was filtered and concentrated under reduced pressure and the residue was purified by recrystallization in hot hexane to afford 1.35 g (70 %) of the 6-chloronicotinic acid active ester intermediate as a white powder [18]. TLC: (EtOAc/hexane 4/1): *R*_*f*_ = *0.28*. ^1^H-NMR (600 MHz, CDCl_3_) δ 7.11 (tt, 1H, J = 7.1 Hz, J = 9.9 Hz), 7.57 (d, 1H, J = 8.3 Hz), 8.43 (dd, 1H, J = 8.3 Hz, J = 2.7 Hz), 9.21 (d, 1H, J = 2.7Hz). *m/z* (ESI) C_12_H_4_ClF_4_NO_2_ ([M + H^+^]) calcd. 305.0, found 304.9.

6-Chloronicotinic acid active ester intermediate (130 mg) [18] was dissolved in 3 mL of a 1 M Me_3_N solution in THF and stirred 2 h at 25 °C. After 5 min, a white precipitate was formed. After completion of the reaction, the precipitate was collected by filtration and washed with diethyl ether and cold CH_2_Cl_2_. The obtained white powder was suspended in 5 mL of CH_2_Cl_2_ containing 2 % TMSOTf and sonicated for 10 min. The reaction mixture was concentrated under reduced pressure and washed with diethyl ether to afford 140 mg (68 % over two steps) of a gray powder after drying. ^1^H-NMR (600 MHz, CD_3_CN) δ 7.43 (tt, 1H, J = 7.4 Hz, J = 10.5 Hz), 8.07 (dd, 1H, J = 8.6 Hz, J = 0.8 Hz), 8.85 (dd, 1H, J = 8.6 Hz, J = 2.3 Hz), 9.34 (dd, 1H, J = 2.3 Hz, J = 0.8 Hz). *m/z* (ESI) C_15_H_13_F_4_N_2_O_2_ ([M^+^]) calcd. 330.1, found 330.0.

#### (*S*)-2-[3-((*S*)1-Carboxy-5-((6-trimethylammonium-pyridine-3-carbonyl)-amino)-pentyl)-ureido]-pentanedioic acid trifluoroacetate salt 9

To a solution of 80 mg (164 μmol) of compound **5** in CH_2_Cl_2_ (4 mL) was added compound **8** (100 mg, 209 μmol) and 100 μL of DIPEA (572 μmol). The reaction was stirred for 2 h at 25 °C and then concentrated under reduced pressure.

Progress of the reaction was monitored by TLC: (CH_2_Cl_2_/MeOH 4/1): *R*_*f*_ = *0.26.* HPLC purification was performed on a semi-preparative Jupiter C12 column (100 Å, 10 μm, 250 × 10 mm). The eluting solvent started with a gradient from 5/95 to 70/30 acetonitrile/(water 0.5 % TFA) for 20 min at a flow rate of 2 mL min^−1^. Then the eluent was kept at 70/30 acetonitrile/(water 0.5 % TFA) for 10 min to elute the desired compound at 25.8 min. After removal of the solvent under reduced pressure gave 96 mg (77 %) of desired compound **9** as a white powder. ^1^H-NMR (600 MHz, D_2_O) δ: 1.32 (s, 9H), 1.34 (s, 9H), 1.35(s, 9H), 1.35–1.39 (m, 2H) 1.55–1.66 (m, 3H), 1.70–1.82 (m, 2H), 1.95–2.03 (m, 1H), 2.30 (M, 2H), 3.36 (t, 2H, J = 6.8 Hz), 3.57 (s, 9H), 4.02 (ddd, 2H, J = 9.5 Hz, J = 8.7 Hz, J = 5.1 Hz), 7.94 (d, 1H, J = 8.8 Hz), 8.35 (dd, 1H J_t_ = 8.8 Hz, J_d_ = 2.3 Hz), 8.57 (d, 1H, J = 2.3 Hz). ^13^C-NMR (125.7 MHz, D_2_O) δ: 23.35, 27.63, 28.19, 28.20, 28.31, 28.78, 31.92, 32.58, 40.80, 54.34, 54.99, 56.06, 83.47, 84.18, 84.36, 115.48, 118.47, 133.37, 141.14, 148.90, 160.16, 167.46, 174.55, 175.14, 175.33. *m/z* (HRMS) C_33_H_56_NO_8_ ([M^+^]) calcd. 650.4123, found 650.4116. Mp = 56 °C.

### Radiosynthesis and quality control of [^18^F]DCFPyL

Radiosynthesis of [^18^F]DCFPyL was performed on a GE TRACERlab^TM^ FX (GE Healthcare, Mississauga, ON, Canada). The synthesis module was modified in terms of program and hardware (see Fig. 3). The synthesis unit was installed and operated in a shielded hot cell.

Analytical HPLC was carried out using a Gilson HPLC (Mandel Scientific Company Inc.; Guelph, Ontario, Canada) by injection of HPLC-purified [^18^F]DCFPyL onto a Phenomenex Nucleosil Luna C18 column (10 μm, 250 × 10 mm) and elution with 20 % CH_3_CN/0.2 % TFA for 5 min at 2 mL min^−1^, followed by gradient elution from 20 % to 38 % CH_3_CN for 5 min and from 38 % to 70 % CH_3_CN for 15 min with isocratic elution at 70 % CH_3_CN for 15 min. Radio-TLC analysis on silica gel plates gave a *R*_*f*_ value of 0.6 in 95 % CH_3_CN/H_2_O (Additional file 1: Figure S4).

### Automated synthesis of [^18^F]DCFPyL

Radiosynthesis of [^18^F]DCFPyL was performed on a GE TRACERlab^TM^ FX (GE Healthcare, Mississauga, ON, Canada). The synthesis module was modified in terms of program and hardware (Fig. 3). The synthesis unit was installed and operated in a shielded hot cell.

### In vivo tumor models

All animal experiments were carried out in accordance with the guidelines of the Canadian Council on Animal Care (CCAC) and approved by the local animal care committee (Cross Cancer Institute, University of Alberta).

PET imaging experiments were carried out in LNCaP and PC3 tumor-bearing Balb/c nude mice (Charles River Laboratories, Quebec, Canada). Male Balb/c nude mice were housed under standard conditions with free access to standard food and tap water. LNCaP and PC3 cells (5 × 10^6^ cells in 100 μL of PBS) were injected into the upper left flank of the mice (20–24 g). Before injecting LNCaP cells, the mice received a 1.0-mg/pellet containing testosterone in a 60-day release preparation (Innovative Research of America, Sarasota, FL, USA).

The pellet was implanted subcutaneously into the upper right flank in order to provide a constant level of testosterone needed by the androgen receptor positive LNCaP cells. Tumors reached sizes of approximately 1 cm^3^ which were suitable for all in vivo experiments.

### Radiometabolite analysis

Normal BALB/c mice were injected with 10–20 MBq of [^18^F]DCFPyL. Venous blood samples were collected at 5, 15, 30, and 60 min p.i. via the mouse tail vein and further processed. Blood cells were separated by centrifugation (13,000 rpm × 5 min). Precipitation of proteins in the supernatant was achieved by the addition of 2 volume parts of MeOH, and the samples were centrifuged again (13,000 rpm × 5 min). Fractions of blood cells, proteins, and plasma were measured in a Wizard gamma counter to determine radioactivity per sample. The clear plasma supernatant was injected onto a Shimadzu HPLC system.

The samples were analyzed using a Phenomenex Luna 10u C18 [2] 100 A, 250 × 4.6 mm column at a constant flow rate of 1 mL min^−1^ and the following gradient with water/0.2 % TFA as solvent A and CH_3_CN as solvent B: 0–7.5 min 20 % B, 7.5–15 min gradient to 90 % B, 15–20 min 90 % B.

### Dynamic PET imaging

General anesthesia of tumor-bearing mice was induced with inhalation of isoflurane in 40 % oxygen/60 % nitrogen (gas flow = 1 mL min^−1^), and the mice were subsequently fixed in prone position. The body temperature was kept constant at 37 °C for the entire experiment. The mice were positioned in a prone position into the center of the field of view. A transmission scan for attenuation correction was not acquired. The mice were injected with 2–10 MBq of [^18^F]DCFPyL (60–150 ng) in 100–200 μL of isotonic NaCl solution (0.9 %) through a tail vein catheter. For blocking studies, the animals were pre-dosed with 300 μg of DCFPyL in 50 μL saline about 10 min prior to radiotracer injection. Data acquisition was performed over 60 min in a 3D list mode. The dynamic list mode data were sorted into sinograms with 53 time frames (10 × 2, 8 × 5, 6 × 10, 6 × 20, 8 × 60, 10 × 120, 5 × 300 s). The frames were reconstructed using maximum a posteriori (MAP) as reconstruction mode. The pixel size was 0.085 × 0.085 × 0.121 mm^3^ (256 × 256 × 63), and the resolution in the center of the field of view was 1.8 mm. No correction for partial volume effects was applied. The image files were processed using the ROVER v 2.0.51 software (ABX GmbH, Radeberg, Germany). Masks defining 3D regions of interest (ROI) were set and the ROIs were defined by thresholding.

Mean standardized uptake values [SUV]_mean_ = (activity/mL tissue)/(injected activity/body weight), mL/g, were calculated for each ROI. Time-activity curves (TACs) were generated for the dynamic scans only. All semi-quantified PET data are presented as means ± SEM. Statistical difference for the blocking study was tested by unpaired Student’s *t* test and was considered significant for *P* < 0.05.

### Internalization experiments

10^5^ LNCaP or PC3 cells were seeded in poly-d-lysine-coated 12-well plates 24–48 h before the assay so that cells could reach 95 % confluency. The medium was removed 1 h before the assay, and the cells were rinsed twice with PBS. After the addition of Krebs buffer (1 mL) to each well, the cells were incubated at 37 °C. Krebs buffer was aspirated, and the cells were incubated with 300 μL of [^18^F]DCFPyL in 0.9 % NaCl (0.1 MBq ) for 60 min at 37 °C. Cellular uptake was stopped by removing incubation media from the cells and washing the wells twice with ice-cold PBS buffer (1 mL). Surface-bound radioactivity was removed from the cells through incubating the cells twice with 0.5 mL glycine-HCl in PBS (50 mM, pH 2.5) for 5 min at 37 °C. Cells were washed again with ice-cold PBS before the addition of radio-immunoprecipitation assay (RIPA) buffer (400 μL) to lyse the cells. Cells were returned into the incubator for 10 min, and cell lysates were collected for counting. Radioactivity of surface-bound and internalized fraction was measured in a WIZARD2 Automatic gamma counter (Perkin Elmer, Waltham, MA, USA). Total protein concentration in the samples was determined by the bicinchoninic acid method (BCA; Pierce, Thermo Scientific 23227) using bovine serum albumin (800, 600, 400, 300, 200, 100, 50 μg/mL, blank) as protein standard. Data are expressed as percent of total uptake per 1 mg protein (% of total uptake/mg protein).

### Tracer kinetic analysis

Tracer kinetic analysis was performed using a two-tissue compartmental model using dynamically acquired PET imaging data. Full details on tracer kinetic analysis are given in the Additional file 1.

## Results

### Chemistry and radiochemistry

**Fig. 2:**
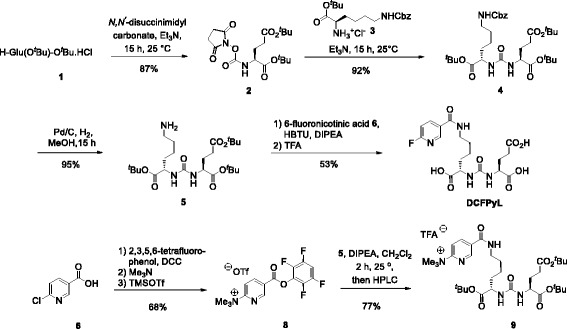
Synthesis of lysine-urea-glutamate peptidomimetics

Synthesis of lysine-urea-glutamate peptidomimetics as highly potent PSMA-binding motifs is given in Fig. 2. Compounds **4**, **5**, and DCFPyL were previously described in the literature [15, 16]. Herein, we present an improved synthesis route [15, 16].

Synthesis commenced with the activation of *tert*-butyl ester protected amino acid H-Glu(O^*t*^Bu)-O^*t*^Bu.HCl **1** with *N*,*N*′-disuccinimidyl carbonate to give corresponding NHS ester (*S*)-(1,5-di-*tert*-butoxy-1,5-dioxopentan-2-yl)carbamic acid **2** in 87 % yield. Coupling of active ester **2** with μ-benzyloxycarbonyl-l-lysine *tert*-butyl ester (H-Lys(Z)-O^*t*^Bu.HCl) **3** afforded 2-(3-{1-*tert*-butylcarboxylate-5-[(carboxybenzyl)-amino]-pentyl}-ureido)-di-*tert*-butyl pentanedioate **4** in 92 % yield. Removal of the *Z* protecting group in compound **4** using hydrogenation on Pd/C gave 2-{3-[1-*tert*-butylcarboxylate-(5-aminopentyl)-ureido}-di-*tert*-butyl pentanedioate **5** in high chemical yields of 95 %.

The free amine in **5** was acylated with 6-fluoronicotinic acid **6** in the presence of HBTU followed by acidic cleavage of *tert*-butyl ester groups using trifluoroacetic acid (TFA) to give reference compound 2-(3-{1-carboxy-5-[(6-fluoropyridine-3-carbonyl)-amino]-pentyl}-ureido)-pentanedioic acid (DCFPyL) in 53 %. The total yield for the four step synthesis of DCFPyL was 40 %.

Trimethylammonium salt **9**, which was the precursor for direct radiofluorination, was prepared by acylation reaction of compound **5** with active ester *N*,*N*,*N*-trimethyl-5-((2,3,5,6-tetrafluorophenoxy)carbonyl)-pyridin-2-aminium triflate **8**. Active ester **8** was prepared with slight modifications based on a published procedure [21] in 68 % yield in two steps starting from 6-chloronicotinic **6** acid. Activation with 2,3,5,6-tetrafluorophenol gave compound **7** which was treated with trimethylamine followed by TMSOTf to afford compound **8**. Compound **9** was obtained through reaction of compound **5** with active ester **8** in 77 % yield after purification by HPLC.

**Fig. 3:**
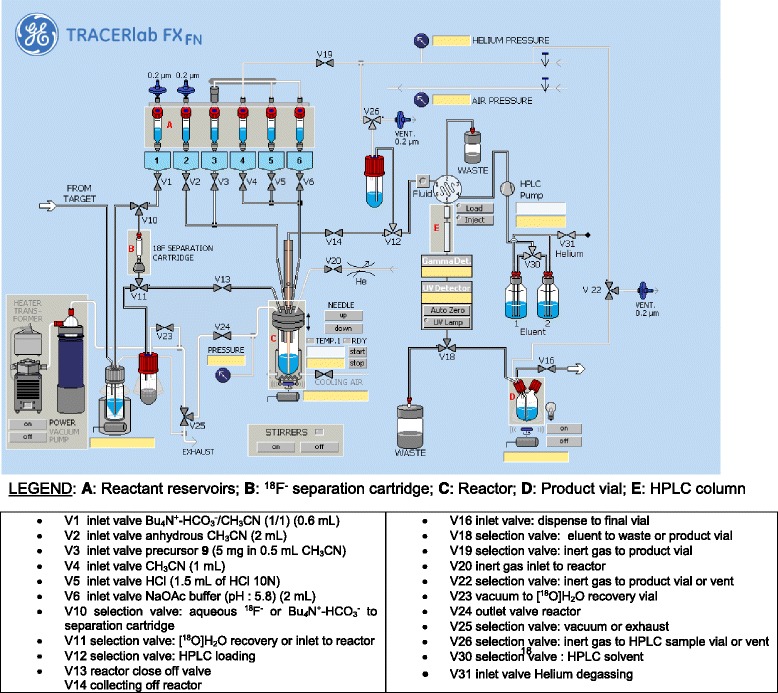
Automated synthesis unit for the radiosynthesis of [^18^F]DCFPyL

Automated radiosynthesis of [^18^F]DCFPyL was performed on a GE TRACERlab^TM^ FX_FN_ automated synthesis unit (ASU) equipped with an integrated HPLC system using a Luna semi-prep HPLC column (Fig. 3).

**Fig. 4:**
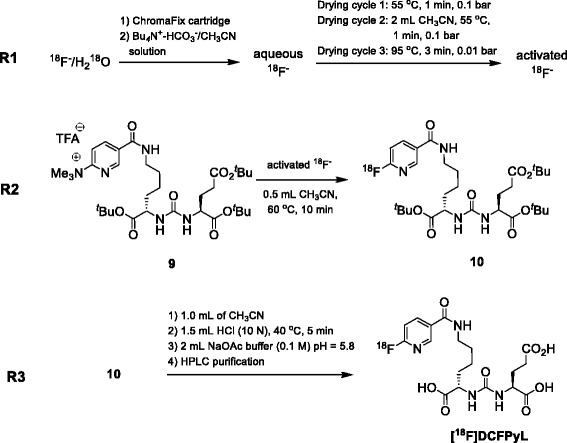
Radiosynthesis of [^18^F]DCFPyL

The overall radiosynthesis of [^18^F]DCFPyL is displayed in Fig. 4, and the optimized HPLC purification is given in the supplementary materials (Additional file 1: Figure S3).

In the first reaction step (**R1**), cyclotron-produced no-carrier added (n.c.a.) [^18^F]fluoride was captured from [^18^O]H_2_O target solution onto a Chromafix cartridge. A solution (0.6 mL) containing Bu_4_N^+^-HCO_3_^−^ (0.3 mL, 0.075 M) and CH_3_CN (0.3 mL) was used to elute n.c.a. [^18^F]fluoride from the resin into the reactor. The aqueous [^18^F]fluoride solution was dried azeotropically through consecutive addition and removal of anhydrous CH_3_CN. The first drying cycle was performed at 55 °C for 1 min at 0.1 bar. After the addition of CH_3_CN (2 mL), solvents were evaporated at 55 °C for 1 min at 0.1 bar followed by an evaporation step at 95 °C for 3 min under high vacuum (0.01 bar). The end of the process yielded activated n.c.a. [^18^F]fluoride suitable for subsequent nucleophilic radiofluorination. This three-step azeotropic distillation allows for minimum dispersion of [^18^F]fluoride on the side of the reactor vial and concentrates most of the radioactivity at the bottom of the reactor. This is important for the subsequent radiolabeling step which uses only a small volume of solvent (0.5 mL).

The second reaction (**R2**) involved combining n.c.a. [^18^F]fluoride and **9** to form compound **10** via a nucleophilic heteroaromatic substitution reaction. Radiofluorination was carried out with 5 mg of labeling precursor **9** in anhydrous CH_3_CN (0.5 mL) at 60 °C for 10 min.

Final reaction step (**R3**) included removal of the *tert.*-butyl ester protecting groups in compound **10** via acidic cleavage using 1.5 mL of HCl (10 N) in 1 mL CH_3_CN at 40 °C for 5 min to give crude [^18^F]DCFPyL.

The pH of the crude reaction mixture was adjusted by the addition of 2 mL of 0.1 M NaOAc (pH 5.8), and the reaction mixture was transferred into a 5-mL injection loop and injected onto a Luna C-18 column (10 μm, 250 × 10 mm) for purification employing isocratic elution with 18 % EtOH containing 0.2 % H_3_PO_4_ at a flow rate of 2 mL min^−1^. Product peak was collected between 22 and 23 min (Additional file 1: Figure S3). The collected peak (~2 mL) was transferred, through a sterile filter, into a sterile vial containing 12 mL of 0.1 M NaOAc (pH 5.8).

The total synthesis time was 55 min, including HPLC purification. The overall isolated radiochemical yield of [^18^F]DCFPyL was 23 ± 5 % (*n* = 10, decay-corrected).

### Quality control of [^18^F]DCFPyL

**Fig. 5:**
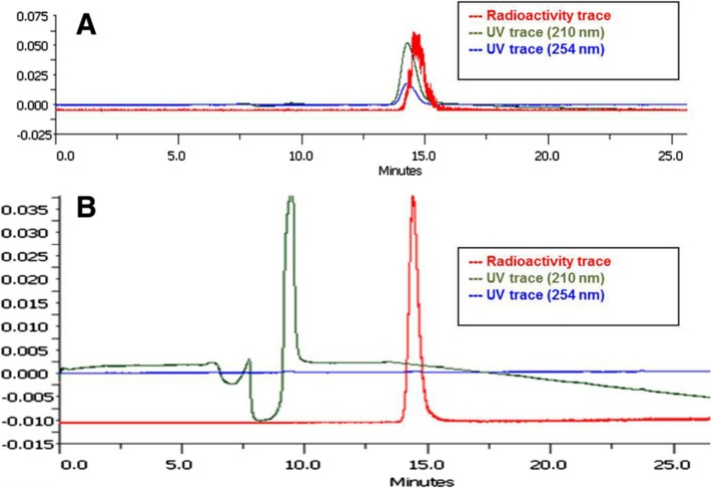
**a** HPLC traces for confirmation of identity after co-injection of [^18^F]DCFPyL with reference compound DCFPyL and **b** quality control of the final product. Detectors are connected in series

Identity of [^18^F]DCFPyL was confirmed by radio-HPLC and radio-TLC using co-injection and co-spotting with reference compound [^19^F]DCFPyL, respectively. Representative HPLC traces are given in Fig. 5a. Quality control revealed high radiochemical purity of 98 %, and the specific activity was determined to be 80–100 GBq/μmol (Fig. 5b, Additional file 1: Figures S5 and S6) starting with 15 to 26 GBq of n.c.a. [^18^F]fluoride. Radio-TLC analysis on silica gel plates gave a *R*_*f*_ value of 0.6 in 95 % CH_3_CN/H_2_O (Additional file 1: Figure S4), and the product was stable (>95 %) in saline of up to 4 h.

### Cell-specific internalization experiments

**Fig. 6:**
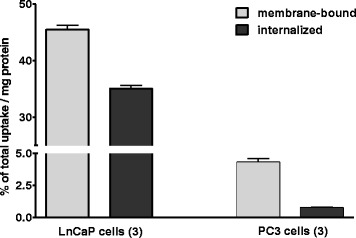
Cell-specific internalization experiments of [^18^F]DCFPyL in PSMA+ LNCaP and PSMA− PC3 cells 60 min after incubation with the radiotracer at 37 °C

Radiotracer [^18^F]DCFPyL revealed substantial higher cell membrane accumulation and internalization in PSMA+ LNCaP cells compared to PSMA− PC3 cells (Fig. 6). After incubation for 60 min, cellular uptake of [^18^F]DCFPyL was stopped and cells were washed with glycine-HCl to remove radioactivity bound to the membrane. Internalized fraction of radioactivity was determined after lysing the cells with RIPA buffer. After 60 min, radioactivity uptake in PSMA+ LNCaP cells reached 45.47 ± 1.01 % of total uptake/mg protein for the membrane-bound fraction and 35.05 ± 0.69 % of total uptake/mg protein for the internalized fraction. This reflects a 44 % fraction of internalized radioactivity in PSMA+ LNCaP cells after incubation with radiotracer [^18^F]DCFPyL for 60 min. In contrast, only 4.30 ± 0.40 % of total uptake/mg protein and 0.76 ± 0.05 % of total uptake/mg protein were found in the membrane-bound and internalized fraction, respectively, in the case of PSMA− PC3 cells.

### Dynamic PET imaging experiments

**Fig. 7:**
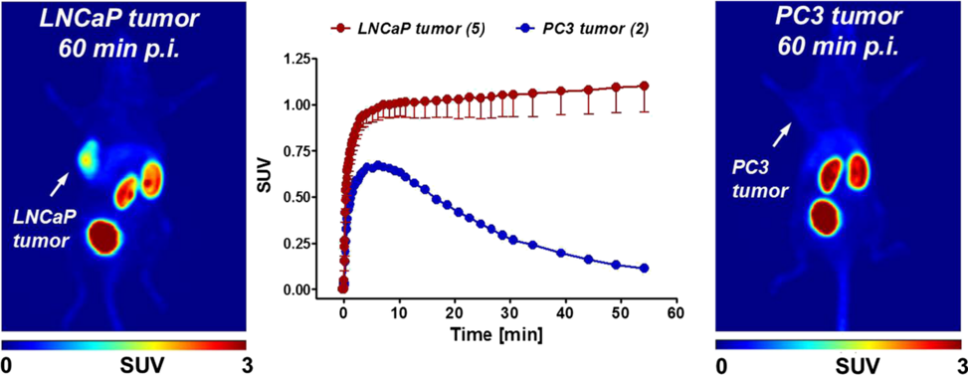
PET images of [^18^F]DCFPyL after 60 min p.i. into LNCaP (*left*) and PC3 (*right*) tumor-bearing BALB/c nude mice. *Middle*: Time-activity curves for radioactivity uptake in LNCaP and PC3 tumor

Radiotracer [^18^F]DCFPyL was injected into PSMA+ LNCaP and PSMA− PC3 tumor-bearing BALB/c nude mice. As shown in Fig. 7, high radioactivity uptake and retention was observed in the PSMA+ LNCaP tumor, whereas only very low uptake and no retention was seen in the PSMA− PC3 tumor. Analyzed TACs describe a continuous increase of radioactivity accumulation and retention in the PSMA+ LNCaP tumor over 60 min reaching a standardized uptake value (SUV_60min_) of 1.1 ± 0.1 (*n* = 5).

In contrast, PSMA− PC3 tumors showed some radioactivity uptake during the perfusion phase followed by rapid washout of radioactivity reaching a SUV of 0.1 (*n* = 2) at 60 min p.i..

In both prostate cancer models, radioactivity is rapidly cleared from blood and muscle. Radiotracer [^18^F]DCFPyL is eliminated through the kidneys with somewhat more retention of radioactivity in the kidneys in the PC3 model.

Only very little radioactivity is found in all other organs resulting in a low background signal. Rapid clearance from blood and muscle tissue led to high tumor-to-blood and tumor-to-muscle ratios of 8.9 ± 1.9 and 17.0 ± 3.4, respectively, after 60 min p.i. in LNCaP-bearing mice.

Specificity of radiotracer [^18^F]DCFPyL for PSMA was demonstrated through blocking experiments in the LNCaP model. LNCaP PSMA+ model was injected with 300 μg of nonradioactive DCFPyL 5 min prior to the administration of radiotracer [^18^F]DCFPyL. Control and blocking experiments were carried out in the same animal on 2 consecutive days.

**Fig. 8:**
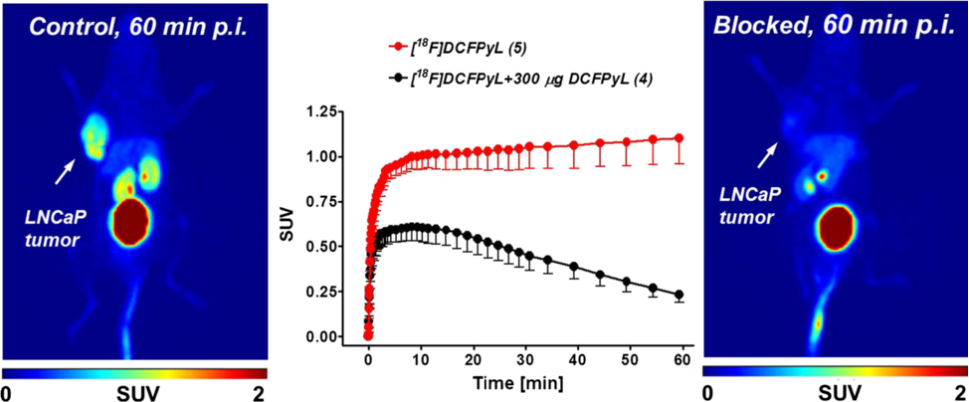
PET images of [^18^F]DCFPyL after 60 min p.i. into a LNCaP tumor-bearing BALB/c nude mouse. *Left*: control; *Middle*: time-activity curves for uptake and blocking in LNCaP tumors in the absence and presence of nonradioactive DCFPyL; *Right*: blocked

Acquired PET images confirmed substantial decrease of radioactivity uptake in the LNCaP tumor, which was statistical significant (*P* = 0.0069). Radioactivity uptake was reduced by 80 % after 60 min p.i. upon pretreatment with DCFPyL (SUV_60min_ control: 1.1 ± 0.1, *n* = 5; SUV_60min_ blocked: 0.2 ± 0.05, *n* = 4). Uptake in the kidneys was also reduced (Fig. 8).

### In vivo metabolic stability of [^18^F]DCFPyL

**Fig. 9:**
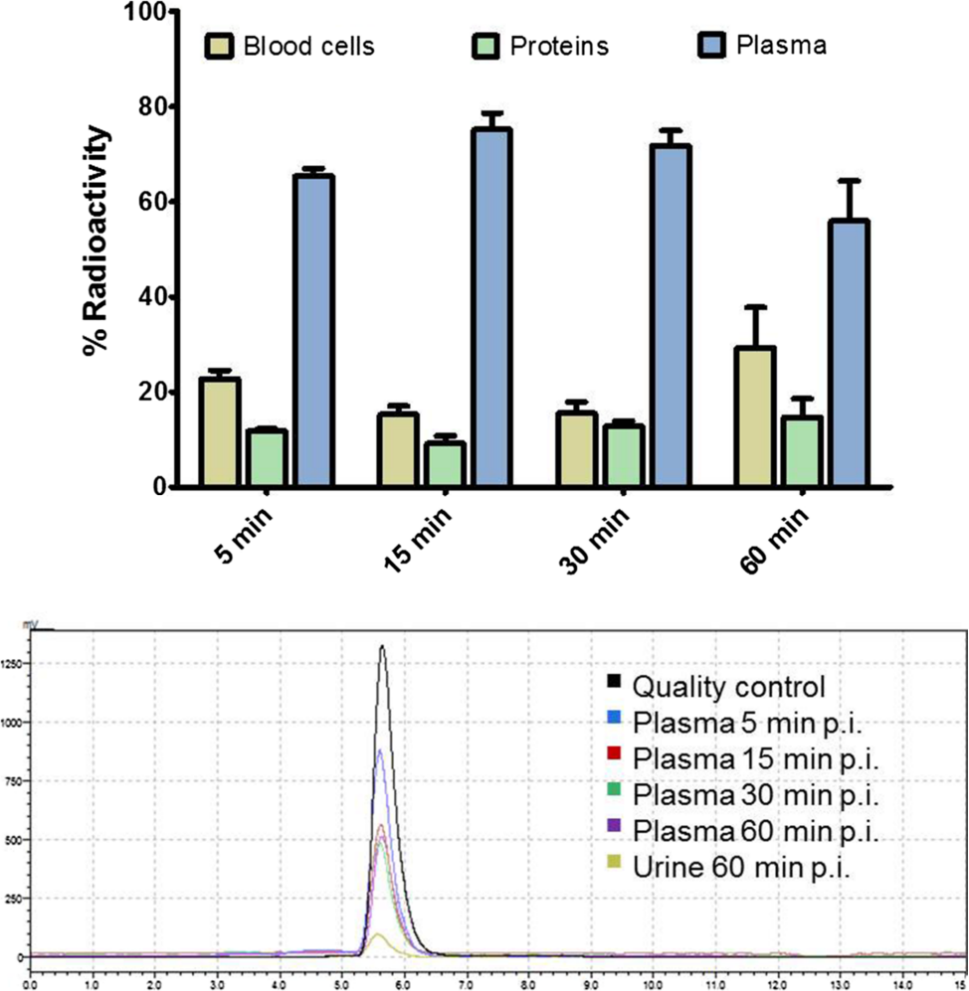
Distribution of [^18^F]DCFPyL in the blood (*top*) revealing only minimal binding to plasma proteins and therefore good bioavailability and evaluation of the metabolic stability of [^18^F]DCFPyL (*bottom*) following injection in BALB/c mice (*n* = 3)

In vivo metabolic stability of [^18^F]DCFPyL was studied by analyzing murine blood samples at different time points and urine after 60 p.i. using radio-HPLC. Figure 9 summarizes the results reflecting the distribution pattern of radioactivity in blood cells, plasma proteins, and plasma over time. The overall distribution of radioactivity in the different blood compartments remained mainly unchanged over time, suggesting rapid equilibration of radioactivity distribution between analyzed blood compartments. The majority of radioactivity is found in the plasma fraction indicating high bioavailability of radiotracer [^18^F]DCFPyL. Radio-HPLC analysis of the plasma samples at different time points confirmed very high metabolic stability of the radiotracer.

High stability of [^18^F]DCFPyL was also found in urine samples after 60 min p.i. In all cases, no radiometabolites were detected in plasma and urine samples over the studied time course of 60 min p.i.

### Tracer kinetic analysis

Cellular uptake of [^18^F]DCFPyL is presumed to follow a two-step process, consisting of first binding to PSMA on the cell membrane and then transport of the [^18^F]DCFPyL-PSMA complex into the cytoplasm. We have therefore modeled the tracer kinetics of [^18^F]DCFPyL using a reversible two-tissue compartmental model. Tracer kinetic analysis was carried out with PET imaging data acquired dynamically from PSMA+ LNCaP and PSMA− PC3 tumor-bearing mice (see Fig. 7). Four kinetic parameters (*K*_1_, *k*_2_, *k*_3_, *k*_4_) describing the flow of the radiotracer from the blood to the tissue (*K*_1_) and from the tissue into the blood (*k*_2_), binding of the radiotracer to the tissue (*k*_3_) and dissociation from the tissue (*k*_4_), were calculated.

**Fig. 10:**
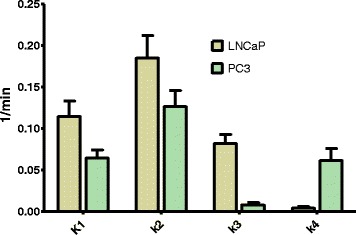
Tracer kinetic analysis of [^18^F]DCFPyL in PSMA+ LNCaP and PSMA− PC3 tumor-bearing Balb/c mice

The analysis was performed by fitting the measured TACs with a two-exponential model of the general form as described, e.g., by van den Hoff (Additional file 1: Figure S2) [22]. Results for the four kinetic parameters are given in Fig. 10.

The net delivery (*K*_1_/*k*_2_) of the radiotracer to the tissue was comparable for LNCaP (*K*_1_/*k*_2_ = 0.62) and PC3 (*K*_1_/*k*_2_ = 0.49) tumors. However, radiotracer [^18^F]DCFPyL accumulated significantly (10.25 times) more in PSMA+ LNCaP tumors compared to PSMA− PC3 tumors (*k*_3_ (LNCaP) 0.082 vs. *k*_3_ (PC3) 0.008). The coefficient *k*_3_/(*k*_3_ + *k*_2_) describes the fraction of radiotracer entering the second compartment (i.e., being bound as opposed to being released back into the blood pool): the value for LNCaP (0.314) dominates that of PC3 (0.058) by more than a factor 5. Significant differences were also found in the dissociation rate (*k*_4_) of the radiotracer from the tumor (*k*_4_ (LNCaP) 0.004 vs. *k*_4_ (PC3) 0.061). Overall, this led to high retention of the radiotracer in LNCaP tumors (*k*_3_/*k*_4_ = 20.5) and negligible retention in PC3 tumors (*k*_3_/*k*_4_ = 0.13).

## Discussion

The present study described the automated radiosynthesis and preclinical validation of (*S*)-2-[3-((*S*)-1-carboxy-5-[3-(6-fluoropyridine)carbonyl)amino)pentyl)ureido]-pentanedioic acid ([^18^F]DCFPyL) as a radiotracer for molecular imaging of prostate-specific membrane antigen (PSMA) in prostate cancer. We performed the radiosynthesis of [^18^F]DCFPyL employing direct nucleophilic heteroaromatic substitution as novel synthesis route in an ASU. The radiopharmacological evaluation of [^18^F]DCFPyL included dynamic PET imaging, metabolic profiling, and tracer kinetic analysis.

The following important results emerged from this study: [1] radiotracer [^18^F]DCFPyL can be prepared in good radiochemical yields suitable for clinical applications via a direct radiofluorination synthesis route in an automated GE TRACERlab^TM^ FX_FN_ synthesis unit; [2] radiopharmacological profile of [^18^F]DCFPyL prepared in an ASU via direct radiofluorination agrees as expected with previously published work such as high specific uptake and retention in PSMA+ tumors, very high metabolic stability, and high bioavailability in vivo*.*

The first part of this study was focused on the development of a novel and automated radiosynthesis of [^18^F]DCFPyL using a direct radiolabeling approach with cyclotron-produced n.c.a. [^18^F]fluoride. Radiochemistry with prosthetic groups for ^18^F labeling represents a special challenge for robust and reliable radiotracer synthesis, especially when automated processes according to GMP guidelines for clinical applications are required. Literature reports on syntheses of ^18^F-labeled small-molecule PSMA inhibitors like [^18^F]DCFPyL mainly involved multiple step reactions using manual synthesis procedures which provided modest to good radiochemical yields [15, 17–19]. Reported decay-corrected radiochemical yields for the preparation of [^18^F]DCFPyL using prosthetic group 2,3,5,6-tetrafluorophenyl-6-[^18^F]fluoronicotinate ([^18^F]FPy-TFP) vary greatly from 5 to 53 % [12, 13, 15]. Low radiochemical yields (decay-corrected) in the range of 5–12 % are reported for [^18^F]DCFPyL when the radiotracer was produced for clinical trials [12, 13]. In one case, the radiosynthesis was carried out in a modified dual-run FDG synthesis module [13]. Higher decay-corrected radiochemical yields of 36–53 % were reported for the original manual synthesis of [^18^F]DCFPyL when modest amounts (1.6–2.2 GBq) of [^18^F]fluoride were used [15]. Various literature reports documented excellent radiochemical yields for direct nucleophilic heteroaromatic substitution reactions with n.c.a. [^18^F]fluoride at the *ortho*-position to prepare various 2-[^18^F]fluoropyridines, including the prosthetic group [^18^F]FPy-TFP, under mild conditions [21]. Recent applications and advantages of this methodology for the design and synthesis of numerous ^18^F-labeled radiotracers have extensively been reviewed [23]. Building on this work, we envisaged a direct radiofluorination synthesis route based on 2-[^18^F]fluoropyridine chemistry for the preparation of [^18^F]DCFPyL using the trimethylammonium salt **9** as the labeling precursor. Attempts to prepare [^18^F]DCFPyL in satisfying radiochemical yields from related 6-chloronicotinic acid compound were not successful (data not shown).

The use of trimethylammonium salt **9** as labeling precursor offers several advantages. Notably as a solid, compound **9** allows for convenient handling and storage. Compound **9** was stored at 4 °C without significant decomposition for at least 3 months. Moreover, excellent leaving group properties of the Me_3_N group in heteroaromatic nucleophilic substitution reactions with n.c.a. [^18^F]fluoride at the *ortho*-position of various pyridines enables radiosynthesis under mild reaction conditions compatible with technology and equipment of ASU like the GE TRACERlab^TM^ FX_FN_ synthesis module.

Automated radiosynthesis of [^18^F]DCFPyL was accomplished in three reaction steps using a one reactor set-up involving drying and activation of cyclotron-produced n.c.a. [^18^F]fluoride (**R1**), incorporation of activated [^18^F]fluoride into compound **10** via nucleophilic heteroaromatic substitution (**R2**), and removal of the *tert*-butyl protecting groups through treatment with HCl and subsequent HPLC purification to give final product [^18^F]DCFPyL (**R3**). The fully automated synthesis provided [^18^F]DCFPyL in decay-corrected radiochemical yields of 23 ± 5 % (*n* = 10) within 55 min, including final HPLC purification. In a typical synthesis, 3 GBq of [^18^F]DCFPyL ready for injection was obtained starting from 20 GBq of n.c.a. [^18^F]fluoride. This is sufficient for several patient doses assuming a single patient dose of 300–400 MBq of [^18^F]DCFPyL. The obtained good and highly reproducible radiochemical yield of 23 ± 5 % clearly demonstrates the favorable features of the direct radiofluorination synthesis route compared to the existing method [12, 13, 15] and should help ensure the availability of the agent. The specific activity was determined to be 80–100 GBq/μM. Radiotracer [^18^F]DCFPyL passed standard quality control test, making module-prepared [^18^F]DCFPyL suitable for human studies.

The second part of this study dealt with the evaluation of module-prepared [^18^F]DCFPyL in preclinical prostate cancer models to further confirm pharmaceutical quality and suitability of the radiotracer for PET imaging of PSMA in vivo.

Dynamic PET imaging of [^18^F]DCFPyL has not yet been reported using PSMA+ LNCaP and PSMA− PC3 tumors. Previously reported studies were using PSMA+ PC3-PIP and PSMA− PC3-flu xenografts [15].

Prostate cancer cell lines LNCaP and PC3 have well-characterized PSMA expression levels to evaluate PSMA radiotracers and are therefore suitable for validation of [^18^F]DCFPyL prepared from the new method in comparison with the original method [15, 17, 18].

As shown in Fig. 6, radiotracer [^18^F]DCFPyL showed high uptake in PSMA+ LNCaP tumors with favorable clearance pattern from blood and muscles. The high uptake and retention of radioactivity in PSMA+ LNCaP tumors correlates with the reported high inhibitory potency of [^18^F]DCFPyL (K_i_ = 1.1 nM) towards PSMA [15]. The fast clearance of the radiotracer from the blood, muscle, and most tissues and organs is in agreement with the hydrophilic nature of compound [^18^F]DCFPyL as typical for peptidomimetic PSMA inhibitors. Uptake of [^18^F]DCFPyL in PSMA+ LNCaP tumors (SUV_60min_ = 1.1) was about 11 times higher compared to PSMA− PC3 tumors (SUV_60min_ = 0.1). After 60 min p.i., only some radioactivity was also found in the kidney with only very minimal background radioactivity in the liver. Most of the injected radioactivity was accumulated in the bladder after 60 min p.i..

This biodistribution profile is consistent with previously reported studies using [^18^F]DCFPyL for PET imaging of PSMA in other prostate cancer models [15]. As expected for PSMA− PC3 tumors, only very little radioactivity was found in the tumor at 60 min p.i.. The highest levels of radioactivity in the PC3 model were also observed in the kidneys and bladder (Fig. 6).

Renal clearance pathway as seen for urea-based peptidomimetic PSMA inhibitor [^18^F]DCFPyL was also reported for ^18^F-labeled phosphoramidate peptidomimetic as small-molecule PSMA-imaging agent in LNCaP- and PC3-bearing mice [18]. Uptake of radiolabeled phosphoramidate in PSMA+ LNCaP tumors was about four times higher compared to the uptake in PC3 tumors as determined by ex vivo biodistribution studies.

Favorable radiopharmacological profile and specificity of [^18^F]DCFPyL for PSMA imaging in vivo was further confirmed by specific blocking studies with nonradioactive DCFPyL using the same animal on 2 consecutive days. The observed 80 % reduction of [^18^F]DCFPyL uptake in PSMA+ LNCaP tumors upon blocking with DCFPyL is indicative of specific targeting of [^18^F]DCFPyL to PSMA.

Metabolic profiling of [^18^F]DCFPyL in mice revealed low binding of the radiotracer to blood cells and plasma proteins leading to high bioavailability. Analyzed plasma samples further confirmed remarkable high metabolic stability of [^18^F]DCFPyL. No radiometabolites were detected by radio-HPLC analysis over 60 min p.i. High metabolic stability was further confirmed by analysis of urine samples at 60 min p.i., which contained only parent compound [^18^F]DCFPyL.

In the last part of this study, we applied tracer kinetic analysis to quantify and compare pharmacokinetics of [^18^F]DCFPyL for PSMA imaging in preclinical LNCaP and PC3 prostate cancer models. Delivery of the radiotracer (*K*_1_) is dependent on blood flow, and our data indicate that radiotracer delivery was elevated for LNCaP tumors. However, net delivery as expressed by the *K*_1_/*k*_2_ ratio was comparable for both tumor models (LNCaP: 0.62; PC3: 0.49).

The observed high uptake and retention of [^18^F]DCFPyL in LNCaP tumors was also reflected by the calculated *k*_3_ values for both tumors. This was further supported by the coefficient *k*_3_/(*k*_3_ + *k*_2_), which was five times higher for LNCaP tumors compared to PC3 tumors.

Kinetic parameter *k*_3_ reflects the binding of the radiotracer to the tissue, and the *k*_3_ value for LNCaP is about 10 times higher than the *k*_3_ value for PC3. Tracer binding is favored in PSMA+ LNCaP tumors, as expressed by the ratio *k*_3_/*k*_4_, and is more than 150 times higher than for PC3 tumors. This led to an 11 times higher uptake of the radiotracer in LNCaP tumors, based on SUV values at 60 min p.i.. The high retention of radioactivity in the LNCaP model can be explained by high affinity binding of the radiotracer to PSMA and internalization of the PSMA-ligand complex. This is supported by the cell-specific internalization experiments in PSMA+ LNCaP and PSMA− PC3 cells. High retention of radioactivity in LNCaP tumors and rapid clearance of radioactivity from most tissues and organs support findings from earlier work on [^18^F]DCFPyL as an ideal radiotracer for PSMA imaging in vivo [15].

## Conclusions

We have developed an automated synthesis for radiotracer [^18^F]DCFPyL based on a direct radiofluorination synthesis route. We demonstrated that [^18^F]DCFPyL can be prepared in good radiochemical yields and pharmaceutical quality suitable for clinical applications for PSMA imaging in humans. The automated synthesis based on direct radiofluorination should improve availability of [^18^F]DCFPyL for PSMA imaging in humans. Dynamic PET imaging of [^18^F]DCFPyL in PSMA+ LNCaP and PSMA− PC3 tumor-bearing mice confirmed the previously reported data describing high PSMA-mediated tumor uptake and favorable clearance profile of the radiotracer. Compartmental model analysis points to a two-step molecular trapping process based on PSMA binding and subsequent internalization leading to retention of radioactivity in PSMA+ LNCaP tumors. These findings further underline the excellent characteristics of [^18^F]DCFPyL for PET imaging of PSMA in vivo.

## Additional file

Additional file: Supplementary information. Automated synthesis of [18F]DCFPyL via direct radiofluorination and radiopharmacological evaluation in preclinical prostate cancer models. (DOCX 446 kb)

## Abbreviations

MAP: maximum a posteriori
PET: positron emission tomography
PSMA: prostate-specific membrane antigen
ROI: regions of interest
SUV: standardized uptake value
SUV: standardized uptake value
TAC: time-activity curve
TLC: thin-layer chromatography
