# Supplementary material for: Automated synthesis of [18F]DCFPyL via direct radiofluorination and validation in preclinical prostate cancer models
Source: EJNMMI Res. 2016 May 4;6:40. doi: 10.1186/s13550-016-0195-6 (PMC4854855; doi:10.1186/s13550-016-0195-6)
Supplement: Additional file: — Supplementary information. Automated synthesis of [18F]DCFPyL via direct radiofluorination and radiopharmacological evaluation in preclinical prostate cancer models. (DOCX 446 kb) [file 13550_2016_195_MOESM1_ESM.docx]

**-Supplementary Information-**

**Automated synthesis of [^18^F]DCFPyL via direct radiofluorination and radiopharmacological evaluation in preclinical prostate cancer models**

Vincent Bouvet^1^, Melinda Wuest^1^, Hans-Soenke Jans^1^, Nancy Janzen^2^, Afaf R. Genady^2^, John F. Valliant^2^, Francois Benard^3^, Frank Wuest^1^

*^1^Department of Oncology, University of Alberta, Edmonton, Canada*

*^2^Department of Chemistry and Chemical Biology, McMaster University, Hamilton, Canada*

*^3^Department of Radiology, University of British Columbia, Vancouver, Canada.*

**Tracer kinetic analysis**

Tracer kinetic analysis was performed using a two-tissue compartmental model using dynamically acquired PET imaging data. A volume of interest (VOI) was defined for the tumor using the Rover software (ABX GmbH, Dresden, Germany) by applying a threshold of 50% SUV_max_ within an ellipsoid mask around the tumor on an image representing the average of the last 40 min of the acquisition (i.e. excluding the tracer uptake phase). Image derived input functions were obtained from regions around the heart, defined by the injected blood bolus visible in the first few time fames of the dynamic acquisitions, using a threshold of 75% of SUV_max_ in that region. Four kinetic parameters (K1, k_2_, k_3_, k_4_) and the fractional blood volume (*fbv*) which accounts for the non-zero vascular space within the tumor ROI, describe the model.

The analysis was carried out by fitting the measured tumor time-activity curves (TACs) with a two-exponential model of the general form:

$TAC_{tumor}=(1-fbv)\cdot\left(A_{1}\exp(-\alpha_{1}t)+A_{2}\exp(-\alpha_{2}t)\right)\otimes TAC_{blood}+fbv\cdot TAC_{blood}$ [1]

where denotes convolution and *fbv*, $A_{1}$, $\alpha_{1}$, $A_{2}$, $\alpha_{2}$ are fit parameters, the latter four representing the amplitude and time dependence of the exponentials (1). They are related to the kinetic parameters reported by Zaidi *et al*. (Figure S1) (2).

**Figure S1.** Fit parameters A_1_, α_1_, A_2_, α_2_ representing amplitude and time dependence of exponential in equation (1)

$$\left.\begin{matrix} \alpha_{1}=0.5\cdot\left(k_{2}+k_{3}+k_{4}-\sqrt{(k_{2}+k_{3}+k_{4})^{2}-4k_{2}k_{4}}\right) \\ \alpha_{2}=0.5\cdot\left(k_{2}+k_{3}+k_{4}+\sqrt{(k_{2}+k_{3}+k_{4})^{2}-4k_{2}k_{4}}\right)\text{ } \\ A_{1}=\frac{K_{1}}{\alpha_{2}-\alpha_{1}}\left(k_{3}+k_{4}-\alpha_{1}\right) \\ A_{2}=\frac{K_{1}}{\alpha_{2}-\alpha_{1}}\left(\alpha_{2}-k_{3}-k_{4}\right) \end{matrix}\right\}$$

$TAC_{tumor}=(1-fbv)\cdot\left(A_{1}\exp(-\alpha_{1}t)+A_{2}\exp(-\alpha_{2}t)\right)\otimes TAC_{blood}+fbv\cdot TAC_{blood}$ [1]

The fit of equation [1] to the experimentally acquired tumor TACs was implemented in Matlab (The Mathworks, Inc., v. R2014b), utilizing a Nelder-Mead simplex direct search. The fit was governed by the minimization of the sum over all time points of square differences between the measured values and the model prediction (Figure S2).

**Figure S2.** Fit of experimentally acquired time-activity curves using equation [1]


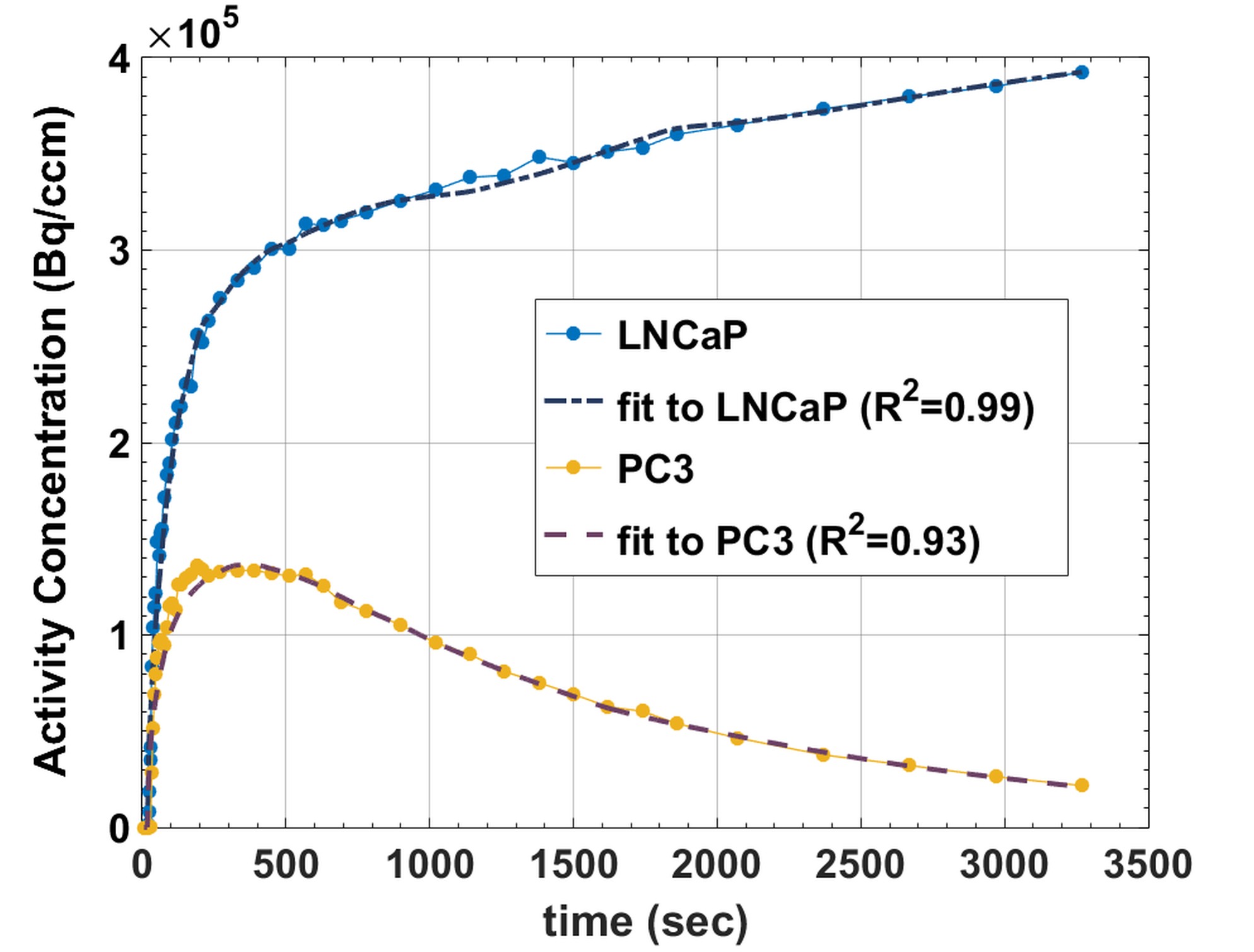


**Figure S3.** Gamma and UV HPLC chromatograms of a typical [^18^F]DCFPyL synthesis


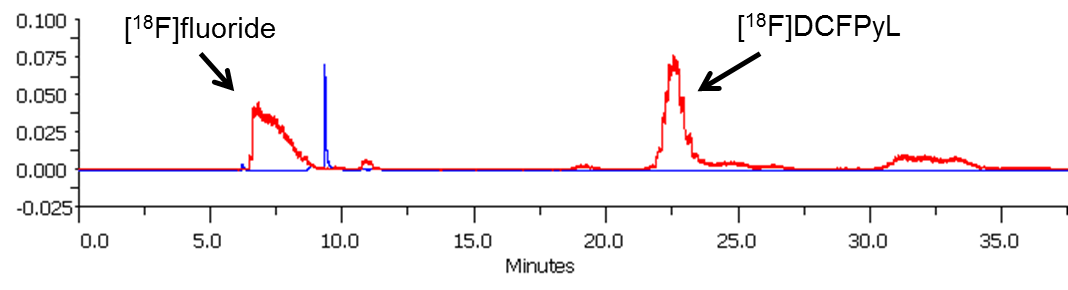


**--- Radioactivity trace**

**--- UV trace (254 nm)**

**Figure S4:** Radio-TLC quality control of [^18^F]DCFPyL.


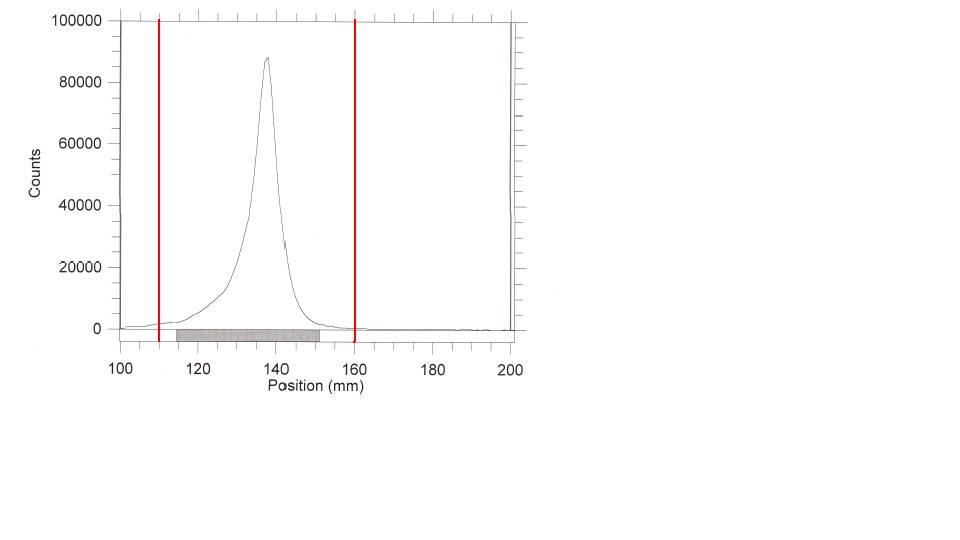


**Figure S5:** Examples of HPLC traces for calibration curve creation for the determination of specific activity

**
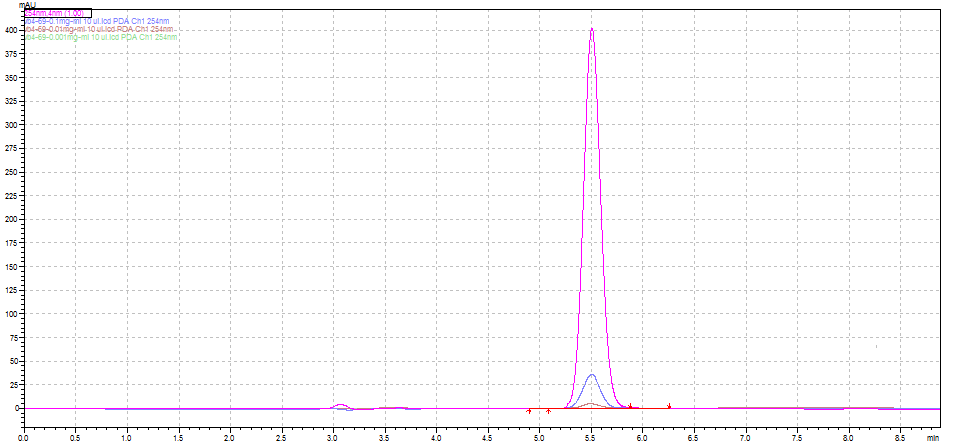
**

1mg.mL^-1^

0.1mg.mL^-1^

0.01mg.mL^-1^

**
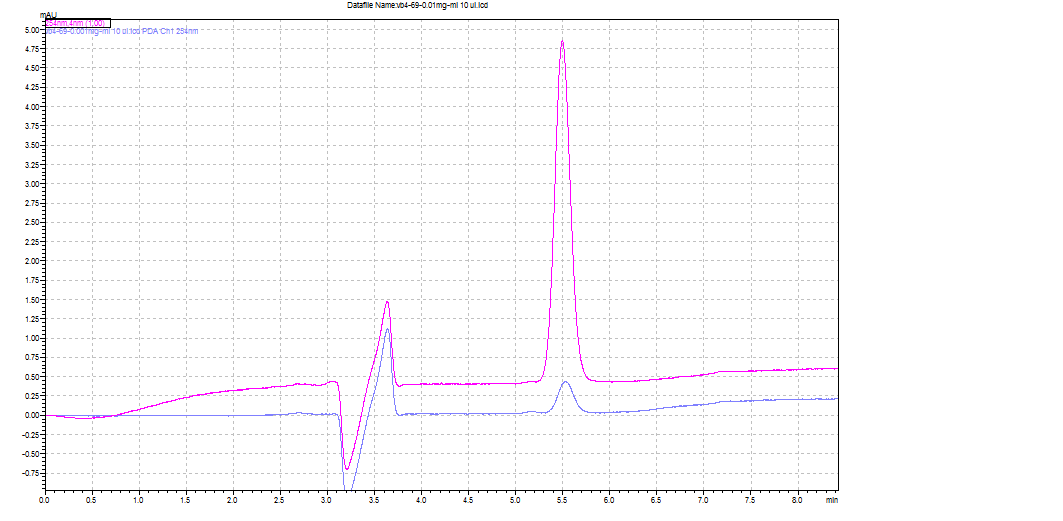
**

0.01mg.mL^-1^

0.001mg.mL^-1^

Each standard HPLC injections of DCFPyL (1 mg/mL - 1 µg/mL) have been repeated 3 times and recorded at 254 nm. The average integrations under the curves with their standard deviation were reported in our specific activity calibration curve. (10 µL injection)

**Figure S6:** Calibration curve for the determination of specific activity

**
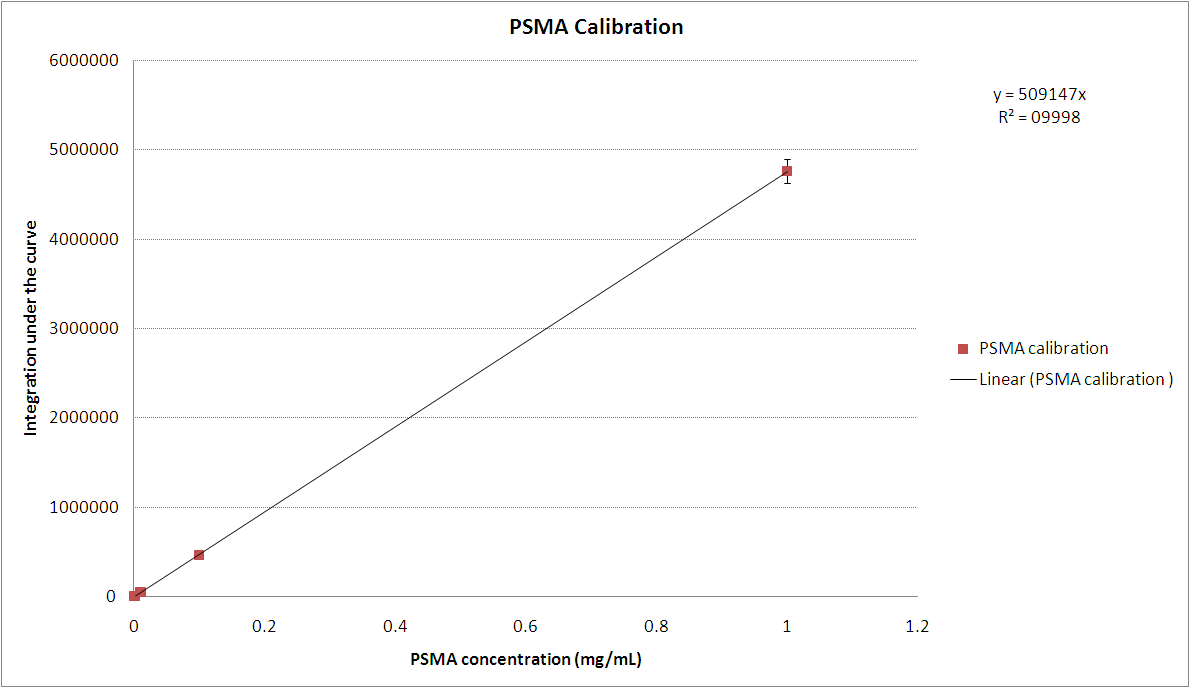
**

With an integration of 1030 (area under the curve), the concentration of [^18^F]DCFPyL (referred to as PSMA in the graph) relates to 0.002 mg/mL. The overall specific activity was determined to be 101 ± 9 GBq/µmol. The average specific activity was determined to be 89 ± 10 GBq/µmol (n = 5) for syntheses ranging from 15 to 26 GBq of n.c.a. [^18^F]fluoride as starting activity.

**References**

1. van den Hoff J. Principles of quantitative positron emission tomography. Amino Acids. 2005, 29:341-353.

2. “Quantitative Analysis in Nuclear Medicine Imaging” by Habib Zaidi (editor), Springer 2006, ISBN-10: 0-387-23854-9.
